# Improving depression and perceived social support enhances overall quality of life among myocardial infarction survivors: necessity for integrating mental health care into cardiac rehabilitation programs

**DOI:** 10.1186/s41983-022-00521-6

**Published:** 2022-07-14

**Authors:** Vivek Upadhyay, Samrat Singh Bhandari, Durga Prasad Rai, Sanjiba Dutta, Pau García-Grau, Krishna Vaddiparti

**Affiliations:** 1 Department of Psychiatry, Sikkim Manipal Institute of Medical Sciences, Sikkim Manipal University, Sikkim 737102, India.; 2 Department of Cardiology, Sir Thutob Namgyal Memorial Multispecialty Hospital, Sikkim 737102, India.; 3 Programa de Maestro de Educación Infantil, Universidad Católica de Valencia, San Vicente, Mártir, Valencia, Spain.; 4 Department of Epidemiology, University of Florida, Gainsville, USA.

**Keywords:** Myocardial infarction, Cardiac rehabilitation, Quality of life, Prevalence, Depression, Social support

## Abstract

**Background:**

Depression and low perceived social support (PSS) have been found to deleteriously affect quality of life (QoL) among myocardial infarction (MI) survivors. The complex relationship between these variables has not been assessed. We wanted to assess first the prevalence of depression among MI survivors and whether depression mediates the effect of PSS on QoL and, second, whether the physical and social domains of QoL mediated the effect of depression and PSS on the emotional domain. This cross-sectional study was done among MI survivors using Cardiac Depression Scale, MacNew Quality of Life After Myocardial Infarction Questionnaire and Multidimensional Scale of Perceived Social Support to assess for depression, QoL and PSS respectively.

**Results:**

A total of 103 MI survivors were included in the study, and the mean age was 59.66 (± 10.42) years. Depression was found in 21.36% of the participants. The indirect effect of PSS on QoL with depression as a mediator was significant (*b* = 0.15, *p* < 0.001, 95% CI 0.12, 0.18). The direct effect of PSS on QoL controlling for depression was also significant (*b* = 0.05, *p* < 0.001, 95% CI = 0.02, 0.07). Depression as a mediator in the relationship explained 75.3% of the effect of PSS on QoL. PSS and depression did not have a significant direct effect on emotional QoL, but it became significant when the physical and social domains were included in the model. The total indirect effects of PSS and depression on emotional QoL were *b* = 0.16, *p* < 0.001, 95% CI = 0.05, 0.17 and *b* = − 0.05, *p* < 0.001, 95% CI = 0.06, − 0.03, respectively.

**Conclusion:**

Depression and poor PSS impair physical and social domains, which impairs the emotional domain of QoL; as such, overall QoL is undermined. As limited physical and social activity because of depression and poor PSS may increase the risk of further cardiovascular events, a holistic approach which includes mental health care is warranted.

## Background

Cardiovascular diseases (CVDs) have become the leading cause of morbidity and mortality among the Indian population [1]. CVDs affect the South Asian population, including Indians, almost a decade earlier than their Western counterparts. The reason for this high tendency to develop CVDs includes the biology of the population and an unhealthy lifestyle of living [1, 2]. This raises the issue of the Asian population, including Indians showing high susceptibility to ischemic heart disease (IHD), and the most common is myocardial infarction (MI). MI occurs when a coronary artery is blocked or almost blocked, which creates a severe reduction in blood flow to some of the heart muscles that experience infarction [3]. During the last few decades, the prevalence of IHD has been increasing at an alarming rate, both in urban and rural populations [1]. When one considers the prevalence of depression following acute MI, almost 50% of patients develop depression, and the rate of major depression is between 15 and 20% [4]. In a meta-analysis performed recently, the pooled prevalence of depression among acute MI patients was found to be 28.70% (95% CI: 22.39–35.46%), and the variability in studies was explained by different factors [5]. Depression results in the hyperactivity of hypothalamic–pituitary–adrenal axis and sympathetic nervous system which contributes to increased heart rate, blood pressure and damage to the arterial system [6, 7]. Other pathophysiological mechanisms which suggest depression increases the risk of CVD include elevated pro-inflammatory cytokines like interleukin-6 [8, 9], enhanced platelet activation [10]. Depression in post-MI patients has been associated with poor adherence, not only to the prescribed treatment for cardiac illness, but also to other comorbid physical illnesses. It has also been related to nonadherence to cardiac rehabilitation measures and poor motivation to make changes in lifestyle that are needed after suffering acute MI [4].

Depression has also been found to be significantly associated with poor quality of life (QoL) in post-MI patients [11]. Patients with cardiac illness having depression are more likely to report more symptom burden, poor quality of life, worse physical limitations and poor overall health when compared to cardiac patients with no depressive symptoms [12]. Patients with IHD and hypertension who have lower QoL have difficulty controlling emotional expression and a lower emotional resistance in challenging situations. Emotional functioning is closely related to an individual’s QoL [13].

The cognitive models of depression given by Beck and colleagues [14] and the hopelessness theory given by Alloy and colleagues [15] explain cognitions can lead to impairment in interpersonal functioning. The idea behind cognitive theory suggests that there is a ‘negative belief with a strong interpersonal focus’. Consistent with this, a depressed individual tends to negatively evaluate himself or herself and also perceives a decline in social support [16]. It was found that elderly individuals with physical impairments reported poor perceived social support (PSS) and poor QoL or life satisfaction [17]. Social support, if not available or if not appreciated by affected individuals, also results in unsatisfactory QoL [18, 19].

As mentioned previously, although past studies have mentioned the link between depression, social support and QoL separately, these variables have not been assessed together to examine their interrelationship, especially in the Indian context. Mediation analysis can help in identifying the complex relationship between the above discussed variables and could explain the process by which the variables affect each other. We conducted a cross-sectional study to examine the association of PSS with QoL and whether depressive symptoms mediate their association. We also wanted to determine how PSS and depression affected the different domains of QoL. We propose the following hypothesis:

*H*_*1*_ A higher perception of social support will lead to better QoL, and depression mediates this relationship.
*H*_*2*_ The physical and social domains of QoL mediate the relationship between both PSS and depression and the emotional domain of QoL.

## Methods

This was a cross-sectional observational study performed at the Department of Cardiology of Sikkim Manipal Institute of Medical Sciences (SMIMS), Sikkim, India. Sikkim is a small state in the northeastern part of India with a population of approximately 700,000. SMIMS was the only center with a facility for intervention for patients with MI. Patients who were diagnosed with acute myocardial infarction (AMI) and were on follow-up between January 2018 and February 2019 were included for assessment. Patients were included for assessment after they had completed the first 3 months following AMI. This time lag of 3 months was taken when the immediate psychological effect of the event was seen as a reaction to the stress of suffering acute MI [20]. Additionally, QoL reaches a steady level at a minimum of 2 months after an episode of MI [21]. Patients who had a history of depression before the episode of myocardial infarction, patients who were critically ill/unstable, and patients who were unable to comprehend and respond to the questionnaires were excluded from the assessment for the study. As depression scores were intended to measure as a mediator (H_1_), the sample size was calculated according to the linear multiple regression. G*Power was used to perform a priori power analysis. Assuming α of 0.05, power of 0.95, effect size 0.15 and with two tested parameters, the total sample size was 89. Sampling was convenient, and we intended to assess all eligible patients during the period of the study. All eligible participants were assessed 3 months after they suffered MI by a psychiatry trainee who had one and half years of experience administering various psychiatric rating scales at the time of conducting the study. The sociodemographic characteristics of the patients were recorded at the beginning of the assessment. They were then assessed with the following assessment tools: The Cardiac Depression Scale (CDS), The MacNew Quality of Life After Myocardial Infarction Questionnaire (MacNew QLMI), Multidimensional Scale of Perceived Social Support (MSPSS).

The Cardiac Depression Scale (CDS) is a 26-item seven-point Likert scale ranging from ‘strongly disagree’ to ‘strongly agree’. A higher score on the CDS indicates greater depression [22]. For diagnosing depression, a cutoff score of 90 was taken based on the findings of Wise and colleagues [23]. The CDS has been validated in different populations, including East Asian and South Asian populations [24]. The MacNew QLMI is a 27-item scale that assesses the ‘social function’, ‘emotional function’ and ‘physical limitation’ domains of health-related QoL. It is a valid, reliable, and interviewer-administered condition-specific health-related quality of life questionnaire. In any domain, the maximum possible score is 7, and the minimum is 1. A higher score on the MacNew QLMI suggests better quality of life [25, 26]. The MSPSS assesses the participants’ perception about the adequacy of social support from three specific sources: family, friends and significant others [27, 28]. Perceived social support is graded as—it consists of 12 items rated on a 7-point Likert scale with scores ranging from ‘very strongly disagree’ (1) to ‘very strongly agree’ (7). A total social support score was calculated for each participant, with higher scores indicating higher perceived social support. The scale has been validated for different ethnic populations as well as specifically the Indian population [29, 30].

The Institutional ethical committee of Sikkim Manipal University approved the study (SMIMS/IEC/520/17–051). The study was conducted according to the criteria set by the Declaration of Helsinki. All participants provided verbal and written informed consent after fully understanding the benefits and risks of participation.

### Statistical analysis

Categorical data were described using counts (*n*, %). The mean and standard deviation (SD) were calculated for continuous variables (age). Correlation analysis was performed to examine the relationship between MSPSS, CDS and MacNew QLMI with sociodemographic variables controlled. Fisher’s exact test was done to find the association between depression status and pattern of substance use (harmful, dependent), number of MI (first episode, ≥ 2 episodes) and presence or absence of medical comorbidity. The CDS score was dichotomized as score ≥ 90 as depressed and < 90 as not depressed [23]. A *p*-value of < 0.05 was considered significant for all the tests. Analysis was performed using SPSS 20 (IBM SPSS Statistics for Windows, Version 20.0. Armonk, NY: IBM Corp.). To determine how depression affects the consequences of PSS in the Mac New QLMI, we performed mediation analysis using JASP software v0.16 (JASP Team, 2021). Assuming that emotional QoL is dependent on both social and physical QoL, we included both depression and social support as the independent variables influencing emotional QoL through the social and physical QoL ratings (mediators). The indirect effect would be significant with CIs not including zero [31]. Bootstrapping of 5000 samples was performed to find the effect in mediation analysis. The sample size adequacy for the mediation analysis (H_2_) was calculated using G*Power 3[32]. A post hoc calculation for multiple regression analysis was employed with a probability value of .05, a medium effect size, and a sample size of *N* = 103. The statistical power for our analysis, considering our *N*, was 1 − β = 0.885, *F*(4, 98) = 2.46, *p* < 0.05, *f*^2^ = 0.15, with four predictors.

## Results

A total of 121 participants who had visited the cardiology outpatient department for follow-up following recovery from acute myocardial infarction were screened. Out of 121 patients, seven patients had a history of depressive episodes before AMI, five patients died during the first 3 months following AMI, and six patients had not given consent for the study. Finally, 103 patients were included in the study. All participants were assessed 3 months following myocardial infarction. The mean age of participants was 59.66 (± 10.42) years. The prevalence of depression was found to be 21.36% (*N* = 22) (CDS score ≥ 90). The sociodemographic details of the participants are given in Table 1. Fisher’s exact test did not find any significant association between depression and the pattern of substance use (*p* = 0.195), number of episodes of MI (*p* = 1.00) and presence of other medical comorbidity (*p* = 1.00).

Correlation analysis showed that QoL Total and the Physical, Social and Emotional domains were negatively correlated with depression and positively correlated with PSS (Table 2).

**Fig. 1:**
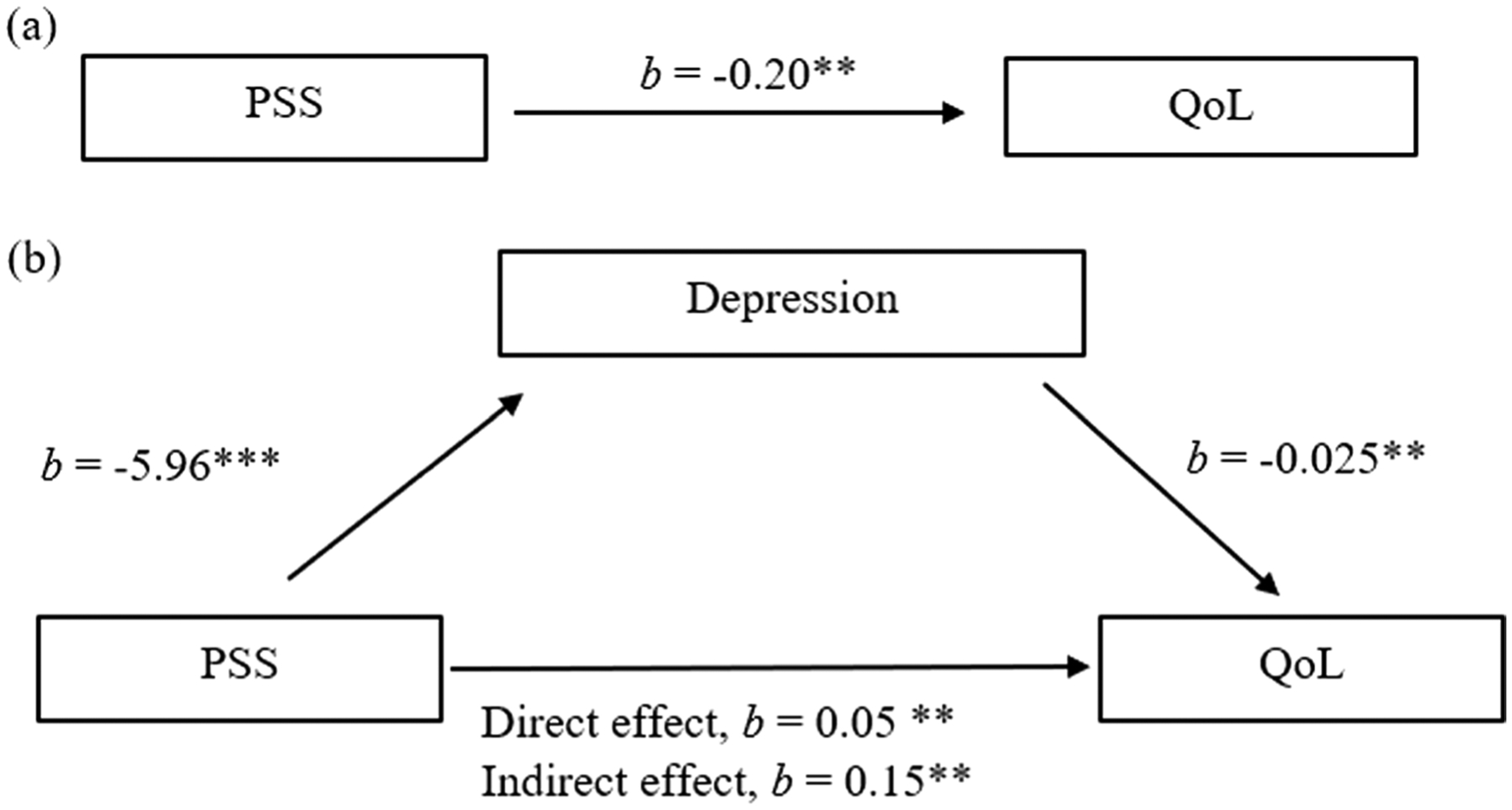
Model of Perceived Social Support predicting Quality of Life, mediated by Depression with Non-standardized estimates. The confidence interval for the indirect effect is a BCa bootstrapped CI based on 5000 samples. **p < 0.001

*H*_*1*_ A simple mediation analysis was performed (Fig. 1) to determine whether depression mediates the relationship between MSPSS and QoL. The MSPSS had a significant effect on depression (*b* = − 5.96, *z* = − 11.74, *p* < 0.001), suggesting that a higher perception of social support meant lower depression. Depression also had a significant effect on QoL (*b* = − 0.02, *z* = − 15.13, *p* < 0.001), which suggests that higher depression leads to poor QoL. The indirect effect of MSPSS on QoL with depression as a mediator was significant (*b* = 0.15, *z* = 10.24, *p* < 0.001). The direct effect of MSPSS on QoL controlling for depression was also significant (*b* = 0.05, *z* = 4.23, *p* < 0.001). Depression as a mediator in the relationship explained 75.3% of the effect of MSPSS on QoL, suggesting partial mediation. Table 3 shows the estimates from the paths of the mediation model.

**Fig. 2:**
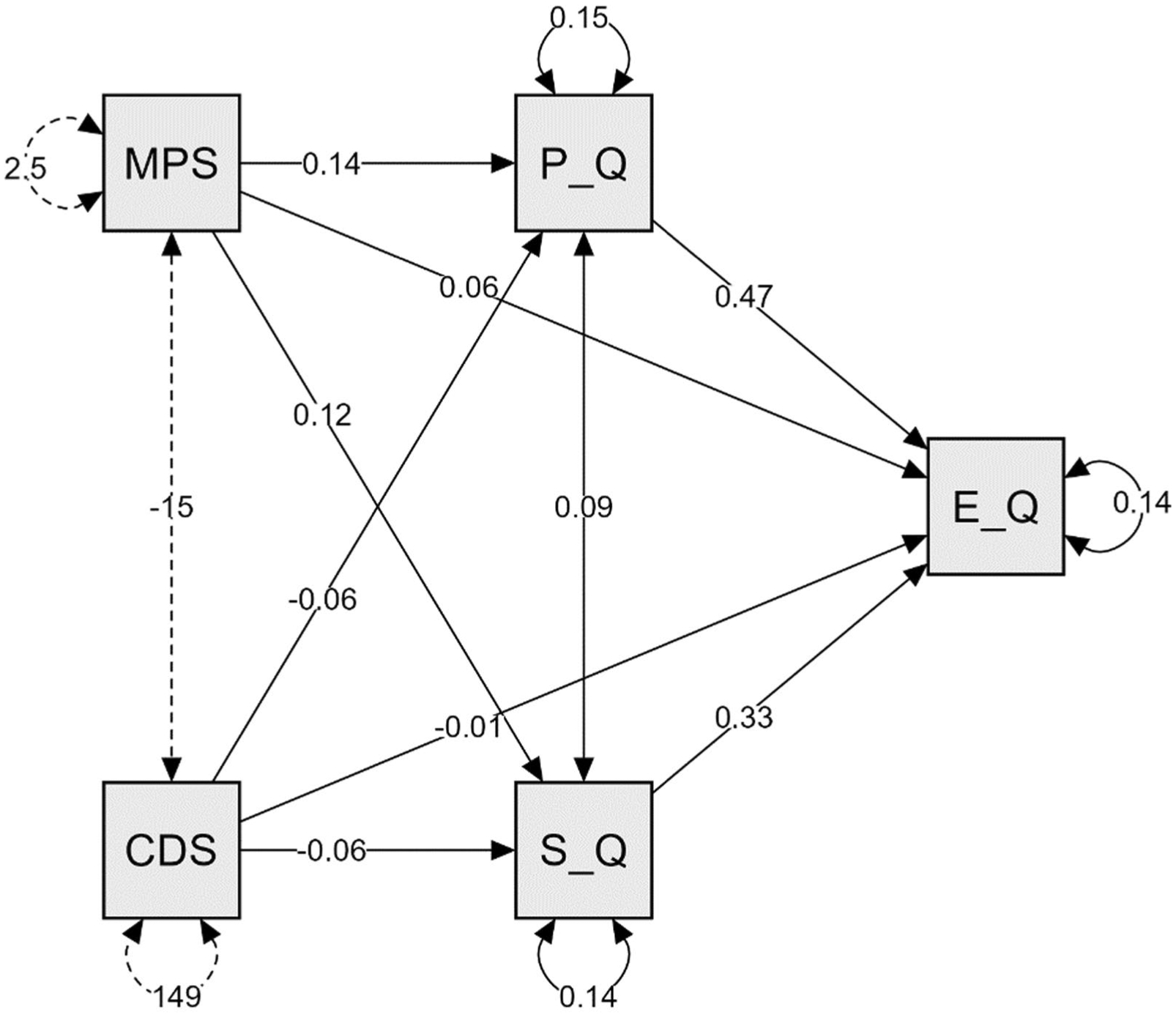
Multiple mediation plot with non-standardized estimates MPS: Multidimensional Scale of Perceived Social Support, CDS: Cardiac Depression Scale, P_Q: Phyical domain of MacNew QoL, S_Q: Social domain, E_Q: Emotional domain

*H*_*2*_ Mediation analyses were conducted to determine the predictive role of social support and depression on emotional QoL, mediated by social and physical QoL (Fig. 2). The results indicated that initially, none of the predictors had a statistically significant effect on emotional QoL, as indicated by the nonsignificant direct effects. However, when mediators were considered in the equation, the indirect effect from MPSSS to emotional QoL was significant through both social (*b* = 0.04, *z* = 2.05, *p* = 0.04) and physical (*b* = 0.07, *z* = 2.63, *p* = 0.008) QoL. These relations were all positive, indicating that the more social support there was, the greater the social and physical QoL perception, which led to a greater emotional QoL. In addition, indirect effects from depression to emotional QoL were also statistically significant, with negative relationships through both social (*b* = − 0.02, *z* = − 2.54, *p* = 0.01) and physical (*b* = − 0.03, *z* = − 3.55, *p* < 0.001) QoL. This negative and significant association indicated that the more depressed participants were, the lower the scores on physical and social QoL and thus the lower emotional QoL.

Total effects showed a statistically significant influence on Emotional QoL, controlling for the mediators. Total effects were significant from MPSSS (*b* = 0.16, *z* = 3.56, *p* < 0.001), with a positive relationship. From Depression, however, the relation was negative and highly significant (*b* = − 0.06, *z* = − 9.34, *p* < 0.001). This result indicated that, controlling for social and physical QoL, depression scores highly predicted lower levels of emotional QoL.

Total indirect effects (with both mediators in the equation at the same time) also indicated statistically significant relations from MPSSS to emotional QoL (*b* = 0.11, *z* = 3.48, *p* < 0.001) and from depression to emotional QoL (*b* = − 0.05, *z* = − 6.60, *p* < 0.001). The direct effects were nonsignificant, and total effects showing relevant predictions indicated a total mediation effect. Table 4 shows the estimates from the different paths of the mediation model.

Figure 2 presents a summary of results in a graphical representation with all non-standardized estimates.

## Discussion

This study highlights how depression and PSS affect the QoL of people who have survived MI. We found depression among 21.36% of our patients. Depression partially mediated the relationship between PSS and QoL. A higher depressive score led to poor perception of the available social support and poor quality of life. Additionally, both depression and poor PSS indirectly and negatively affected the emotional domain of QoL, with the physical and social domains as mediators.

The mean age of our participants was 59.66 (± 10.42) years, and the current prevalence of depression in a similar age group population was found to be 3.53% in the National Mental Health Survey, 2015–16 [33]. Furthermore, the World Health Organization gave the global estimate of depression and stated that the prevalence of depression in the 55- to 74-year-old age group was 7.5% for females and 5.5% for males [34]. Our finding for depression after MI in the same age group was 21.36%, which is much higher than both the national and global figures and is in line with the findings of the meta-analysis reported by Feng and colleagues, who reported a pooled prevalence of 28.7% (95% CI: 22.39–35.46) [5], and that of Thombs and colleagues who reported a prevalence of 19.8% (95% CI: 19.1–20.6%) [35]. Post MI patients with clinical depression have been found to have an almost twofold increased risk of new cardiovascular events and mortality [36], and the reasons are unhealthy lifestyles, nonadherence to prescribed drugs, diet and exercise programs and irregular follow-up with their physicians, which are more common in depressed patients than in nondepressed patients [37].

As our first hypothesis predicted, we found that depression mediates the effect of PSS on QoL, which is in agreement with the findings of studies performed in patients with human immunodeficiency virus infection and acquired immune deficiency syndrome [38] and elderly patients with multiple morbidities [39]. Wicke and colleagues, in their study on elderly patients with multiple morbidities [39], provided a theoretical explanation that also holds true for our study. The explanation is based on cognitive appraisal theory by Richard S. Lazarus. When an individual faces a stressful event (in our study, MI), there is a primary appraisal of the stressor and a secondary appraisal of the coping resources. Depressive mood may result from a negative primary appraisal of threat and harm caused by MI, coupled with a perceived lack of social support in the secondary appraisal phase. On the other hand, a positive social support perception could counteract a negative primary appraisal. The direct effect of PSS on QoL was significant, controlling for depression, which suggests that regardless of the level of depression, the perception of social support contributed to a better QoL. The presence of social support, though it may be perceived incorrectly by the patient, helps in maintaining a healthy lifestyle and adherence to the prescribed treatment regimen.

We found that depression and PSS did not directly affect the emotional domain of QoL, but when the physical and social domains were included as mediators, the effect became significant. This finding can be explained by the fact that MI survivors’ physical and social activities are limited because of the illness itself and because of lack of motivation and poor confidence in performing physical activity while exercising or engaging in social activity, which can be explained by depression. Our finding is supported by the finding of Nicholson and colleagues who found that MI survivors who have depression have higher odds of having increased body mass index, less physical activity and smoking than those without depression [40]. Furthermore, if the survivors perceive a poor social support and lack of someone who can be around while doing the activities, they may harbor fear of experiencing further injuries, which can lead to avoidance of getting involved in physical activities and social interactions. This limitation negatively affects the emotional domains of QoL. Coull and colleagues, in their qualitative study, also found that MI survivors found that many participants tended to avoid physical exercise when there was nobody around and that social support was a significant facilitator of physical activity [41].

This study has its strength and limitations. It is the first to assess the relationship between PSS, depression and QoL in MI survivors using tools validated among post-MI patients. We used the CDS for assessing depression and the MacNew QLMI for QoL, which are specific for patients in post-MI period, and to the best of our knowledge, none of the Asian studies used both scales together in their study. Second, as the center where the study was performed was the only center with intervention cardiology in the state, almost all the patients who suffered MI were referred to this center, implying a better representation of the population. Furthermore, the sample size was also adequate to test our hypothesis. As this is a cross-sectional study, a causal relation cannot be established, and it would be helpful if the relationship model used in the present study can be tested longitudinally over time among MI survivors. Other limitations of the study includes that we did not take into account the severity of cardiac illness or other medical illness and the current use of medications which may contribute to the depression and poor QoL which our participants were experiencing.

## Conclusions

Psychiatric illnesses are increasingly being recognized as a consequence of physical illnesses. As such, any discipline of medical sciences, that are the parent department, taking care of the affected patient will find improvement in overall outcome if the psychiatric and psychosocial part is simultaneously taken care of. Our research found that patients following MI may develop depression, which decreases the perception of available social support and impairs QoL. We also found that depression decreases the physical and social domains of QoL, which ultimately impairs emotional QoL. If intervention is started for depression on time, it will improve PSS and with actual mustering of social support, the QoL will improve and there will be a lesser chance of re-experiencing a subsequent MI. In countries such as India, rehospitalization may be a huge burden for the family. A secondary prevention approach will be highly recommended where a regular screening of MI survivors for depression and intervention targeting depression and mobilization of social support is recommended. A holistic approach for treating MI survivors, which includes cardiologists, psychiatrists, psychologists, social workers and physiotherapists, will not only result in better outcomes, but will also be cost-effective.

## Figures and Tables

**Table 1:** Sociodemographic characteristics of the participants

Variables	Categoies	*N*	Percentage
Gender	Male	79	76.7
	Female	24	23.3
Education	No formal education	16	15.5
	Attended school	70	68
	Graduate or above	17	16.5
Marital status	Married	97	94.2
	Single/divorced	6	5.8
Socio-economic status	Upper SES	36	35
	Middle SES	67	65
Religion	Hindu	69	67
	Christian	12	11.7
	Buddhist	22	21.4
Substance use	Alcohol	51	49.5
	Tobacco	52	50.5
Pattern of substance use	Harmful pattern	32	31.1
	Dependence pattern	71	68.9
Family type	Nuclear family	83	80.6
	Joint family	20	19.4
Medical comorbidity	None	23	22.3
	Present	80	77.7
Number of episodes of MI	1 episode	100	97.1
	2 or more	3	2.9

**Table 2:** Correlation matrix of relationships among the study variables

Variable	1	2	3	4	5	6	Mean (SD)
1. CDS	–						86.93 (12.25)
2. MSPSS	− 0.775***	–					5.19 (1.59)
3. QoL Total	− 0.929***	0.799***	–				5.12 (0.40)
4. QoL Emotional	− 0.872***	0.778***	0.963***	–			5.25 (0.42)
5. QoL Physical	− 0.913***	0.797***	0.972***	0.918***	–		5.12 (0.41)
6. QoL Social	_—_ 0.919***	0.790***	0.976***	0.912***	0.950***	–	5.08 (0.41)

*CDS* Cardiac Depression Rating Scale, *MSPSS* Multidimensional Scale of Perceived Social Support, *QoL* quality of life.

*** *p* < 0.001

**Table 3:** Mediation with perceived social support as a predictor, quality of life as a dependent variable and depression as a mediator with unstandardized estimates

Mediation estimates							
Effect	Estimate	SE	95% confidence interval		*z*	*p*	% Mediation
			Lower	Upper			
Indirect	0.1528	0.0147	0.1239	0.1811	10.39	<0.001	75.3
Direct	0.0502	0.0127	0.0255	0.0782	3.95	<0.001	24.7
Total	0.2030	0.0157	0.1695	0.2321	12.90	<0.001	100.0

Path estimates							
	Estimate	SE	95% confidence interval		*z*	*p*
			Lower	Upper		
MSPSS → CDS	− 5.9664	0.50045	− 6.8537	− 4.8853	− 11.92	< 0.001
CDS → QoL Total	− 0.0256	0.00176	− 0.0290	− 0.0221	− 14.55	< 0.001
MSPSS → QoL Total	0.0502	0.01271	0.0255	0.0782	3.95	< 0.001

The confidence interval for the indirect effect is a BCa bootstrapped CI based on 5000 samples

**Table 4:** Serial multiple mediation with unstandardized parameter estimates

	Estimate	SE	*z*	*p*	95% CI	
					Lower	Upper
Direct effects						
MSPSS → QoLEmo	0.055	0.039	1.416	0.157	− 0.021	0.131
CDS → QoL Emo	− 0.006	0.008	− 0.728	0.467	− 0.022	0.010
Indirect effect؛						
MSPSS → QoL Soc → QoL Emo	0.041	0.020	2.045	0.041	0.002	0.080
MPSSS → QoL Phy → QoL Emo	0.066	0.025	2.634	0.008	0.017	0.114
CDS → QoL Soc → QoL Emo	− 0.021	0.008	− 2.540	0.011	− 0.037	− 0.005
CDS → QoL Phy → QoL Emo	− 0.028	0.008	− 3.546	<0.001	− 0.044	− 0.013
Total effects						
MSPSS → QoL Emo	0.161	0.045	3.563	<0.001	0.073	0.250
CDS → QoL Emo	− 0.055	0.006	− 9.339	<0.001	− 0.066	− 0.043
Total indirect effects						
MSPSS → QoL Emo	0.106	0.031	3.483	< 0.001	0.046	0.166
CDS → QoL Emo	− 0.049	0.007	− 6.600	< 0.001	− 0.064	− 0.034

The confidence interval for the indirect effect is a BCa bootstrapped CI based on 5000 samples. *Qol Emo* QoL emotional domain, *QoL Soc* QoL social domain, *QoL Phy* QoL physical domain

## Data Availability

Authors will provide the data on reasonable request.

## Abbreviations

PSS: Perceived Social Support
QoL: Quality of Life
CDS: Cardiac Depression Scale
MacNew QLMI: MacNew Quality of Life After Myocardial Infarction Questionnaire
MSPSS: Multidimensional Scale of Perceived Social Support
